# Epidemiological Characterization and the Impact of Healthcare-Associated Pneumonia in Patients Admitted in a Northern Portuguese Hospital

**DOI:** 10.3390/jcm10235593

**Published:** 2021-11-28

**Authors:** Lucía Méndez, Pedro Castro, Jorge Ferreira, Cátia Caneiras

**Affiliations:** 1 Pneumology Department, Centro Hospitalar de Entre Douro e Vouga, 4520-221 Santa Maria da Feira, Portugal; jorge.ferreira@chedv.min-saude.pt; 2 EnviHealthMicro Lab, Microbiology Research Laboratory on Environmental Health, Institute of Environmental Health (ISAMB), Faculty of Medicine, Universidade de Lisboa, 1649-028 Lisboa, Portugal; ccaneiras@medicina.ulisboa.pt; 3 Intensive Care Unit, Centro Hospitalar de Entre Douro e Vouga, 4520-221 Santa Maria da Feira, Portugal; pedro.castro@chedv.min-saude.pt; 4 Institute of Preventive Medicine and Public Health, Faculty of Medicine, Universidade de Lisboa, 1649-028 Lisboa, Portugal; 5 Microbiology and Immunology Department, Faculty of Pharmacy, Universidade de Lisboa, 1649-003 Lisboa, Portugal

**Keywords:** pneumonia, healthcare-associated pneumonia, epidemiology, hospitalization, *Klebsiella pneumoniae*, gram-negative, Portugal

## Abstract

Pneumonia is one of the main causes of hospitalization and mortality. It’s the fourth leading cause of death worldwide. Healthcare-associated infections are the most frequent complication of healthcare and affect hundreds of millions of patients around the world, although the actual number of patients affected is unknown due to the difficulty of reliable data. The main goal of this manuscript is to describe the epidemiological characteristics of patients admitted with pneumonia and the impact of healthcare-associated pneumonia (HCAP) in those patients. It is a quantitative descriptive study with retrospective analysis of the clinical processes of 2436 individuals for 1 year (2018) with the diagnosis of pneumonia. The individuals with ≤5 years old represented 10.4% (*n* = 253) and ≥65 were 72.6% (*n* = 1769). 369 cases resulted in death, which gives a sample lethality rate of 15.2%. The severity and mortality index were not sensitive to the death event. We found 30.2% (*n* = 735) individuals with HCAP and 0.41% (*n* = 59) with ventilator-associated pneumonia (VAP). In only 59 individuals (2.4%) the agent causing pneumonia was isolated. The high fatality rate obtained shows that pneumonia is a major cause of death in vulnerable populations. Moreover, HCAP is one of the main causes of hospital admissions from pneumonia and death and the most pneumonias are treated empirically. Knowledge of the epidemiology characterization of pneumonia, especially associated with healthcare, is essential to increase the skills of health professionals for the prevention and efficient treatment of pneumonia.

## 1. Introduction

Pneumonia, along with other lower respiratory tract infections, is the fourth leading cause of death worldwide, accounting for over 4 million deaths per year [1,2]. At the European Union, pneumonia remains the most frequent cause of death from infection, especially in the elderly [3]. In Portugal, pneumonia is one of the main causes of hospitalization and mortality. In 2018, about 40,345 patients were hospitalized with the diagnosis of pneumonia and the associated mortality rate was 20.3% [4].

Nosocomial infections are infections acquired by a patient during healthcare that did not have it or was not incubating it at the time of admission [5]. They constitute the most frequent complication of health care, but the actual number of patients affected is unknown due to the difficulty of reliable data [6]. These infections increase hospital stay, dysfunctions and promote greater resistance of microorganisms to antimicrobials. Healthcare-associated pneumonia (HCAP) includes any patient who was hospitalized in an acute care hospital for two or more days within 90 days of infection; resided in a nursing home or long-term care facility; received recent intravenous antibiotic therapy, chemotherapy, or wound care within the past 30 days of the current infection; attended a hospital or hemodialysis clinic; or lives with a family member infected with a multidrug resistant organism [7,8]. Within HCAP we find the pneumonia acquired by the Hospital (HAP) and the pneumonia associated with the respirator (VAP). HAP is a pneumonia that occurs 48 h or more after admission which was not incubating at the time of admission [7,8,9]. The definition of VAP is a type of pneumonia acquired in hospitals that occurs more than 48 h after endotracheal intubation. It can be more precisely classified as early onset (until the first 96 h of mechanical ventilation and late onset (more than 96 h after initiation of mechanical ventilation [8,9].

In Portugal, the epidemiological and clinical evidence available is focused on community-acquired pneumonia (CAP) [10,11,12,13,14,15,16,17] at global and regional level in mainland Portugal [18], on CAP and influenza hospitalizations [11,19] and, recently on organizing pneumonia due to COVID-19 [20,21,22]. No studies have focused specifically on HCAP. In fact, epidemiological data to characterize HCAP are scarce and difficult to obtain, despite the relevance for the scientific knowledge and for prevention and therapeutic optimizing of HCAP. The purpose of this manuscript is to describe the epidemiological characteristics of patients admitted with pneumonia and to evaluate the impact of HCAP within the universe of patients admitted with pneumonia.

## 2. Materials and Methods

This manuscript describes a descriptive and quantitative study of all individuals hospitalised for pneumonia in a secondary care hospital in northern Portugal during 2018. All individuals admitted to the hospital from 1 January 2018 to 31 December 2018, with the diagnosis of pneumonia, were included. All hospital admission wards were considered. Epidemiological and clinical variables were analysed, namely: age, gender, pathogenic agent isolated, nosocomial pneumonia, severity index and mortality.

The individuals included in the sample were classified as pneumonia, according to the Homogeneous Diagnostic Groups (HDG), according to the All Patient Refined DRGs (APR-DRG) which is a classification system for patients admitted to acute hospitals that incorporate severity of illness. In order to make this grouping, the International Classification of Diseases 9th Clinical Modification of Review (CIE-9-CM) is used in Portugal [23]. Considering the differences in patients with respect to the severity of the disease and the risk of mortality, this diagnostic grouping allows subdivision into subclasses according to these factors. The severity of the disease is understood as an extension of physiological decompensation or loss of organic function and is subclassified as 4 (1: Minor, 2: Moderate, 3: Major and 4: Extreme). The risk of mortality is understood as the patient’s probability of death and is subdivided into 4 subclasses (1: Minor, 2: Moderate, 3: Major and 4: Extreme) [24,25].

The classification of the HCAP used was based on the Consensus Document on Nosocomial Pneumonia [5]. The guarantee of confidentiality of the information and the anonymity of the participants was a concern in this study, so that in the data collection and analysis process, there was no element that could identify the individuals in the sample. The study was conducted according to the guidelines of the Declaration of Helsinki, and approved by the Ethics Committee of Centro Hospitalar de Entre Douro e Vouga (protocol code CA-102/2020-0t_MP/AC, 24 April 2020).

## 3. Results

Between 1 January 2018 and 31 December 2018 were hospitalized 17,176 individuals. Of these, were admitted with the diagnosis of pneumonia 2436 individuals, representing 14.18% of the total number of individuals admitted in that year.

### 3.1. Epidemiological Characterization

The occurrence of the episodes were more frequent during the coldest months with a total of 61.78% (Autumn: 19.58%, *n* = 477) and Winter: 42.20%, *n* = 1028) than during the more temperate months 38.21% (Spring: 21.84%, *n* = 532) and Summer: 16.38%, *n* = 399). In the sample we found 51.8% (*n* = 1262) men and the mean age was 68.8 ± 27.6 years and median 79 years. Of these 11.9% (*n* = 292) were children of whom 10.38% (*n* = 253) were 5 years old or younger. For individuals over 65 years of age, this corresponds to 72.62% (*n* = 1769) of the sample.

**Figure 1:**
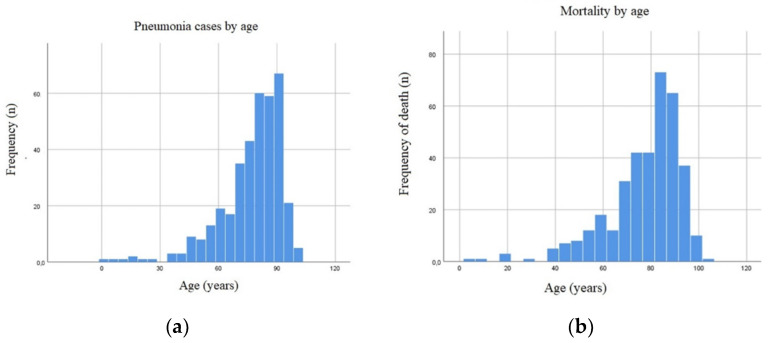
Number of cases of pneumonia (**a**) and mortality (**b**) frequency distributed by age.

The average length of stay was 10.1 ± 7.2 days, the minimum length of stay was 1 day and the maximum length was 81 days. Of the individuals studied 369 resulted in death, which gives a lethality rate of 15.15% (CI 95%; 13.59–16.67). Among the dead individuals we found 0.04% (CI 95%; 195.72–195.80) (*n* = 1) <5 years and 11.08% (CI 95%; 87.99–111.81) (*n* = 270) >65 years which detailed frequency is presented at Figure 1. The individuals were classified acco-rding to the severity and mortality risk index, following the criteria of the Diagnosis Related Groups (DRG) [25] as it is shown in Table 1.

**Figure 2:**
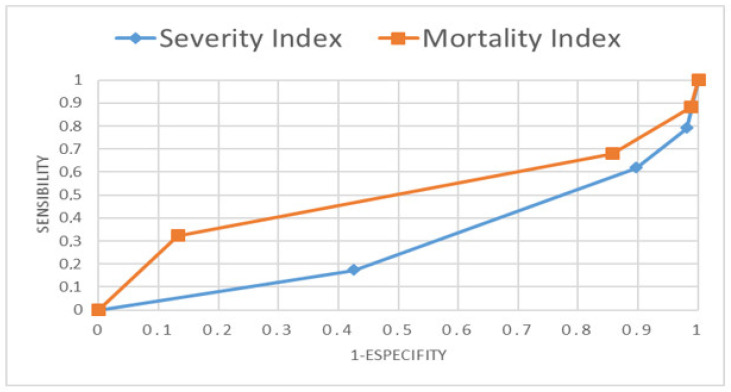
ROC curves of the severity and mortality index as a predictive factor for the occurrence of death.

To determine the degree of specificity and sensitivity of the severity and mortality rates in relation to death prediction, the Receiver Operating Characteristic curves (ROC curves) were performed and analyzed, as can be seen in Figure 2.

### 3.2. Microbiological and HCAP Characterization

**Figure 3:**
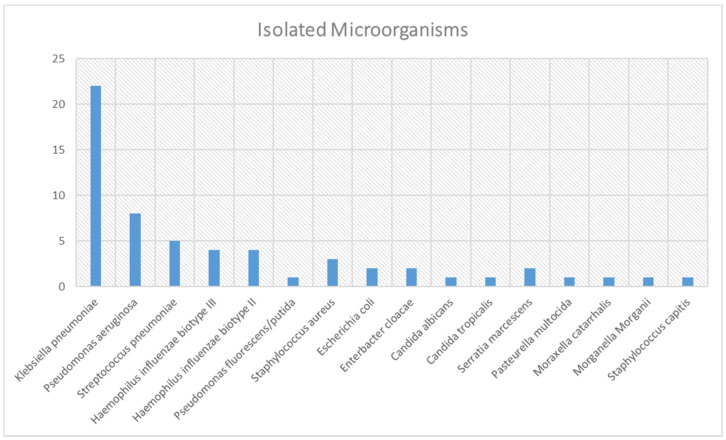
Identification of the microorganisms isolated in respiratory samples.

At only 59 of the individuals the agents causing pneumonia were isolated, which constitutes 2.42% of the total sample. At 39.28% (*n* = 22) of these isolations the Gram-negative pathogen *Klebsiella pneumoniae* was identified (Figure 3). *Pseudomonas aeruginosa* (*n* = 8) was the second most frequently isolated microorganism.

**Figure 4:**
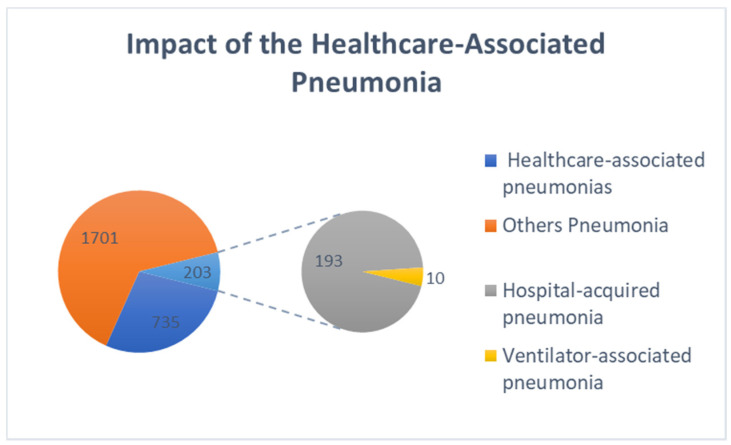
Impact of Healthcare-Associated Pneumonia. This figure represents the number of individuals with HCAP, HAP and VAP, in relation to the total number of individuals admitted for pneumonia.

Considering the 2436 individuals studied with pneumonia, 30.17% (*n* = 735) of their pneumonia were associated with health care. Within these individuals, it was found that in 0.41% (*n*= 10) the pneumonia was associated with mechanical ventilation (VAP) and in 7.92% (*n* = 193) of the cases the pneumonic episode started during the current hospitalization (HAP). Data is shown in Figure 4. Of the 735 individuals with HCAP, 153 died, which is 6.28% of mortality considering all patients with pneumonia, 20.82% of mortality considering the HCAP classification and 41.46% of the total of deaths at hospital at the same period.

Mortality was also analysed for each of the different types of nosocomial pneumonia and related to gender. Of the total number of individuals affected by VAP 0.69% (*n* = 7) in relation to the total sample died, being 0.16% (*n* = 4) males and 0.12% (*n* = 3) females. In relation to individuals with HAP, there were 1.72% (*n* = 42), being 0.78% (*n* = 19) males and 0.94% (*n* = 23) females. The rest of the nosocomial pneumonias fell under HCAP and we found that 6.28% (*n* = 153) died during hospitalisation, with 2.91% (*n* = 71) being male and 3.37% (*n* = 82) being female. These data are shown in Table 2.

## 4. Discussion

The main goal of this study was to characterize the epidemiological and clinical characteristics of the patients admitted with pneumonia at a secondary care hospital in Portugal and to evaluate the impact of HCAP. To the best of authors knowledge, it is the first study focused on HCAP in Portugal.

Pneumonic episodes can occur at any time of the year, they have a greater incidence in the coldest months, especially in winter. The colder air acts as an irritant to the airways, which facilitates the installation and multiplication of infectious agents, and during the colder seasons, people tend to stay longer in closed environments, which favors the propagation of infectious agents among people [26,27]. The gender of individuals is not a predisposing factor for pneumonia, affecting both genders equally. The days of hospital admission obtained in our study corresponds to the average number of days of admission by pneumonia in Portugal [28,29,30]. Of relevance, the most vulnerable population with the highest associated risk of pneumonia is almost the entire sample (over 65 and 5 years old or younger), so being very young or elderly can be a risk factor for pneumonia. Moreover, we demonstrated an age-dependency on the pneumoniae frequency and mortality.

The health system is fundamental for the success of the treatment and prevention of pneumonia. Moreover, it is essential to ensure access to vaccination for vulnerable populations, as well as to encourage healthy lifestyles, with adequate nutrition and, above all, to encourage breastfeeding in children. The characteristics of our current societies, such as increased environmental pollution and overcrowding, may be aggravating factors for the increase of pneumonic episodes in vulnerable individuals, as well as the increased life expectancy in developed countries [2]. Healthcare-associated pneumonia (HCAP) has been introduced as an entity in the ATS/IDSA guidelines update from 2005 [31] and still is a controversy concept, especially in Europe [32,33,34] considering the difficulty to identify predictors for such risk [35].

Mortality from pneumonia is improving in most EU countries, however substantial variation in trends remains between countries and Portugal was excluded due to missing data. [36]. The mortality rate obtained in our study (20.82% of mortality considering the HCAP classification) is similar to previous studies on CAP mortality (20.4%, 2000–2009) [17] and total pneumonia (20.3%, 2018) in Portugal [4] but significantly lower when compared to the data reported in 2015 (57.7%) [3]. Some factors that occurred in the last years in Portugal may have helped reduce this rate. In July 2015, the 23-valent pneumococcal polysaccharide (BPPV23) vaccine was included in the National Vaccination Plan [37,38]. Coincidentally, in 2013 the priority health program was created: Program for the Prevention and Control of Infections and Antimicrobial Resistance (PPCIRA), which aims to reduce the rate of infection associated with health care and promote the correct use of antimicrobials [39]. This mortality rate was particularly worrying in the elderly population, in line with a national study that have concluded that patients older than 75 years and comorbidities contribute decisively to the risk of dying from pneumonia in the hospital [40].

Nosocomial pneumonias have a major impact on hospital admissions, constituting a significant number in relation to the total number of complicated pneumonias requiring hospital admission, with a high risk of death from pneumonia [6]. This implies high health and human costs for hospitals. The quality and safety of care provided in hospitals must be a priority for national healthcare systems. Worryingly, most pneumonia (>95%) are treated empirically considering that only in few situations the infectious agent is isolated. At the top of the list of isolations we find *Klebsiella pneumoniae*, which can indicate that the trend of respiratory isolations has changed in the last years and we can highlight the increase of relevance of Gram-negative microorganisms [41]. However, this results should be interpreted with caution regarding that only 2% of the clinical situations have a microorganism identified.

Of relevance, pneumonia has represented >40% of the total of deaths at the hospital at the same period, highlighting the need to implement more studies on HCAP epidemiology and clinical burden. Previous studies highlighted that room for improvement in antibiotic prescription in Healthcare-Associated Pneumonia currently remains and that new strategies for a better use of the adopted tools and definition of new antimicrobial stewardship initiatives are needed to improve compliance to recommendations [42]. In fact, local guidelines and recommendations to treat common infectious diseases are a cornerstone of most Antimicrobial Stewardship programs [43]. More precise instruments are needed to heighten clinicians’ index of suspicion for treating probable resistant pathogens with appropriate empirical antibiotic choices [42]. Furthermore, an effective surveillance system to provide quality data and improve the monitoring and epidemiologic characterization of Healthcare Associated infections should be evaluated and implemented [44,45,46,47,48].

The Portuguese health institutions code patients with the severity and risk of mortality rates. However, at the clinical level they are not good predictive indicators for the event of death. Their use is more relevant for the management teams of healthcare institutions and standard indices such as Pneumonia severity index or CURB 65, which are suggested by guidelines for pneumonia from American Thoracic Society and Infectious Diseases Society of America [49] are not currently used.

One limitation that this study is the absence of classification of all types of pneumonia that could give us more detailed information about the causes that determine complicated pneumonia that needs hospital admission. Another limitation found is the absence of the antibiotherapy used, which would give us the therapeutic tendency used in the empirical treatment of pneumonia. Overall mortality should be higher in patients who attended a hospital or hemodialysis clinic or received intravenous chemotherapy in the 30 days before pneumonia, and among patients who resided in a nursing home or long-term-care facility [34]. Given the focus on HCAP, information on the Healthcare setting frequented by these patients would be useful, as well as the delay of onset of the HCAP and unfortunately was not available. However, this study provides a robust epidemiological characterization of pneumonia, especially HCAP. To the best authors knowledge this is the first study in Portugal that addressed HCAP epidemiology and clinical burden. Future research is needed to increase knowledge about other types of pneumonia and the relation with causative agents and the treatment used, to better understand the impact that pneumonia on the hospital population.

## 5. Conclusions

This manuscript highlight that pneumonia constitute a high risk of mortality for vulnerable populations and is one of the leading causes of hospital admission. The impact of HCAP alerts us to the critical action of infection prevention and control measures in hospitals and should incentive the increase of scientific studies in this crucial area. Reducing the incidence of pneumonia in general and HCAP in particular, will allow an significative improvement in the quality and safety of patient healthcare.

## Figures and Tables

**Table 1:** Classification according to the severity and mortality rates that follows the DRG criteria [25].

Index	Severity (*n*)	%	Mortality Risk (*n*)	%
1	286	11,74	445	18.27
2	530	21.76	386	15.85
3	1363	55.92	1092	44.83
4	252	10.34	508	20.85
Unclassified	5	0.21	5	0.21

**Table 2:** Classification of the types of nosocomial pneumonia and mortality rate, in total number and discriminated by gender and type of pneumonia considering the total of individuals with pneumonia (*n* = 2436).

Pneumonia	Individuals *n* (%)		Mortality *n* (%)	
		Total	Male	Female
VAP	10 (0.41)	7 (0.29)	4 (0.16)	3 (0.12)
HAP	193 (7.92)	42 (1.72)	19 (0.78)	23 (0.94
HCAP	532 (21.84)	104 (4.27)	48 (1.98)	56 (2.30)
Total	735 (30.17)	153 (6.28)	71 (2.91)	82 (3.37)
